# Is There a Difference in Postoperative Pain After Harvesting a Palatal Soft Tissue Graft Aiming for Primary Versus Secondary Intention Healing? A Systematic Review and Meta‐Analysis

**DOI:** 10.1002/cre2.70476

**Published:** 2026-10-25

**Authors:** Natalia Asquino, Rafael Esteves Lima, Ernesto Andrade, Adriana Drescher, Magdalena Mayol, Kristina Bertl, Andreas Stavropoulos

**Affiliations:** ^1^ Department of Periodontology, Faculty of Dentistry University of the Republic Montevideo Uruguay; ^2^ School of Dentistry Federal University of Minas Gerais Belo Horizonte Brazil; ^3^ Private Practice Montevideo Uruguay; ^4^ Department of Periodontology, Dental Clinic, Faculty of Medicine Sigmund Freud University Vienna Austria; ^5^ Department of Periodontology Blekinge Hospital Karlskrona Sweden; ^6^ Periodontology, Faculty of Odontology Malmö University Malmö Sweden; ^7^ Division of Conservative Dentistry and Periodontology, University Clinic of Dentistry Medical University of Vienna Vienna Austria; ^8^ Department of Periodontology, School of Dental Medicine University of Bern Bern Switzerland

## Abstract

**Objectives:**

To evaluate the effect of surgical technique on postoperative pain at the palatal donor site after harvesting a soft tissue graft.

**Material and Methods:**

A literature search with the following eligibility criteria was conducted: English language; (randomized) controlled clinical trials (RCT/CT); assessment of pain (including morbidity and discomfort), either directly or via surrogate measures, at the palatal donor site; and comparison of two harvesting techniques (i.e., harvesting a soft tissue graft either involving primary [PIH] or secondary intention healing [SIH]).

**Results:**

Five RCTs and two CTs (sample size: 12–50 patients) providing information on a total of 200 donor sites and with a follow‐up period of 1–4 weeks were included. Pain was assessed with different methods, for example, using a visual analog scale, the proportion of patients reporting pain, and/or the quantity of analgesics consumed. Secondary outcomes included complete epithelialization, stress, inability to chew, effects on eating habits, sensitivity disturbances, and burning sensation. Four out of seven studies reported significant differences regarding pain, all favoring the PIH technique. Further, four out of seven studies contributed to a meta‐analysis, which did not show any significant differences in postoperative pain between the SIH and PIH groups at 3 or 7 days. Subgroup analyses including only studies without palatal wound protection also showed no significant differences (*p* ≥ 0.43).

**Conclusions:**

Current evidence does not confirm that measures aiming for PIH when harvesting a palatal soft tissue graft result in significantly less pain at the donor site compared to a technique involving SIH. However, this conclusion should be taken with care due to considerable heterogeneity among the included studies (e.g., different graft/wound dimensions, use/no use of palatal wound protection), the limited number of available RCT, and the observed inconsistency regarding reporting of factors that may influence postoperative pain (e.g., residual palatal tissue thickness).

**Trial Registration:**

The review was registered in the International Prospective Register of Systematic Reviews (PROSPERO; CRD42023416685).

## 1 Introduction

The connective tissue graft (CTG) is considered as the gold standard in root coverage procedures when thickening of the gingiva is required (Chambrone et al. 2008, 2010; Chambrone and Tatakis 2015; Oates et al. 2003; Roccuzzo et al. 2002; Scheyer et al. 2015; Zuhr et al. 2014), as well as for corrections of peri‐implant soft tissue deficiencies or defects (Zuhr et al. 2014; Jung et al. 2022; Jensen et al. 2023; Thoma et al. 2021). Different techniques have been developed to harvest a CTG from the palate. The standard procedure is harvesting a subepithelial CTG (S‐CTG) with a split‐thickness flap approach (Edel 1974; Harris 1997; Huerzeler and Med 1999), which allows primary intention healing (PIH) (Huerzeler and Med 1999). More recently, it has been suggested to harvest the CTG without a flap procedure, either as a free gingival graft (FGG) that is de‐epithelialized then extraorally, or by intraoral de‐epithelialization and then collection of the CTG, that is, a de‐epithelialized free gingival graft (D‐FGG) (Sullivan and Atkins 1968; Couso‐Queiruga et al. 2023; Zucchelli et al. 2010; Tavelli et al. 2019). While extraorally a scalpel blade is used for de‐epithelialization of the graft, various methods have been described for intraoral de‐epithelialization, such as use of a diamond bur, laser, bone scrapers, and so forth (Zucchelli et al. 2010; Sebaoun et al. 2022; Lin et al. 2018; Bara‐Gaseni et al. 2024). This approach leaves an open wound at the palate, and healing occurs by secondary intention, which is considered to increase patient morbidity (Zucchelli et al. 2010; Tavelli et al. 2019; Farnoush 1978). However, a split‐thickness flap approach also includes aspects that might increase patient morbidity compared to a D‐FGG procedure. Specifically, S‐CTG harvesting requires a deeper palatal dissection to preserve flap viability, resulting in a thinner residual palatal tissue layer. The latter has been shown to be associated with increased postoperative pain as well as failure to preserve flap viability, that is, occurrence of flap necrosis (Zucchelli et al. 2010; Burkhardt et al. 2015).

From a clinical perspective, the success of root coverage, the mimicry with the surrounding soft tissue, and the long‐term stability of the results have been validated in the scientific literature with a CTG in general (Cairo et al. 2020; Bertl et al. 2021; Chambrone et al. 2022). Nevertheless, it has been suggested that a coronally advanced flap (CAF) + D‐FGG provides superior clinical results compared to a CAF + S‐CTG (Tavelli et al. 2019), in terms of percentage of root coverage, recession depth reduction, keratinized tissue (KT) gain, and clinical attachment level (CAL) gain (Tavelli et al. 2019). On the other hand, a D‐FGG might lead to a higher prevalence of late complications compared to a S‐CTG, such as the formation of cyst‐like cavities located in the connective tissue releasing a white‐yellowish secretion either spontaneously or after pressure/palpation (Cardoso et al. 2021); this complication is most likely related to epithelial remnants in the graft (Couso‐Queiruga et al. 2023).

In this context, next to the clinical parameters, patient‐reported outcome measures (PROMs) should also be considered (Cairo et al. 2020; Tonetti et al. 2025). Regardless of the harvesting technique, it is known that the palatal donor site adds significantly to the postoperative morbidity (Thoma et al. 2023). Harvesting a S‐CTG with a split‐thickness flap approach aims to reduce patient morbidity due to complete wound coverage and PIH (Edel 1974; Harris 1997; Huerzeler and Med 1999); however, the evidence to support this claim is inconclusive (Farnoush 1978; Jahnke et al. 1993) and—as mentioned above—PIH or secondary intention healing (SIH) might not be the only relevant aspect contributing to postoperative morbidity. The aim of the present systematic review was to summarize the available evidence on the effect of the graft harvesting technique resulting either in PIH or SIH on postoperative pain at the palatal donor site.

## 2 Methods

### 2.1 PICOS Question

The following research question (PICOS) (Miller and Forrest 2001) was defined: “In patients undergoing a soft tissue augmentation procedure around natural teeth or dental implants with the use of a soft tissue graft, does the harvesting technique aiming either for PIH or SIH impact on PROMs (e.g., postoperative pain) at the palatal donor site?”

- Patients: Patients undergoing a soft tissue augmentation procedure around natural teeth or dental implants with a soft tissue graft.
- Intervention: Soft tissue augmentation with a graft harvested from the palate.
- Comparison: Use of a palatal graft aiming either for PIH or SIH at the donor site.
- Outcomes:
  ◦ Primary outcome parameter: Postoperative pain perception at the donor site (including the terms morbidity and discomfort).
  ◦ Secondary outcome parameters: Wound healing at the donor site, sensitivity disturbances, and effect on eating habits due to discomfort from the donor site.
- Study design: Randomized controlled clinical trials (RCTs) and non‐randomized controlled clinical trials (CTs).

### 2.2 Protocol, Registration, and Eligibility Criteria

Reporting of the present systematic review followed the criteria of the Preferred Reporting Items for Systematic Reviews and Meta‐analyses (PRISMA) (Page et al. 2021). The protocol was registered prior to conducting the systematic review in the International Prospective Register of Systematic Reviews (PROSPERO; CRD42023416685). Studies were included if they met the following eligibility criteria:

- RCT or CT.
- Assessment of postoperative pain at the palatal donor site (including the terms morbidity and discomfort), either reported or based on surrogate parameters (i.e., analgesic consumption).
- Comparison of two soft tissue harvesting techniques from the palate either aiming for PIH or SIH.
- No language restriction.
- No restrictions on the follow‐up period or minimum number of participants.
- No restrictions on the handling/treatment or postoperative measures at the donor site.

### 2.3 Information Sources and Literature Search

The electronic literature search was conducted up to February 28, 2026, in the following databases: MEDLINE/PubMed, Embase, CENTRAL, Web of Science, and Scopus. The search strategy was initially developed for PubMed and subsequently adapted for the other databases (Appendix Tables A1, A2, A3). In addition, ongoing and unpublished studies were searched through ClinicalTrials.gov. Gray literature was explored through Google Scholar by screening the first 100 records of each predefined search strategy. Furthermore, forward citation searching was performed using the Science Citation Index, and the reference lists of all included studies and relevant systematic reviews were manually screened for additional eligible studies.

### 2.4 Study Selection

All references retrieved from the different databases were managed with a specific program designed for performing systematic reviews (Rayyan) (Ouzzani et al. 2016). Following removal of duplicates, two authors (N.A. and M.M.) independently screened all titles and abstracts to select the articles according to the above defined eligibility criteria. Whenever more information was required than was available in the abstract, the full text of the article was read. If an article was excluded after reading the full text, the reason for exclusion was recorded, and the authors' agreement at the abstract and full‐text level was calculated (Cohen's kappa). In case of disagreement, a third author (R.E.L.) was consulted to reach consensus by discussion. One author (N.A.) repeated the literature search and screening process.

### 2.5 Data Collection and Extraction

Three authors (N.A., M.M., and E.A.) extracted independently twice the following data: author(s), year of publication, study design, country, sample size per group, patient characteristics, graft harvesting technique, characteristics of the donor site, graft characteristics, postoperative care, postoperative pain (primary outcome), and other postoperative parameters regarding wound healing at the donor site, sensitivity disturbances, any effect on eating habits due to discomfort at the donor site (secondary outcomes), and follow‐up period. Further, any information on any funding, such as self‐supported, research grant, and/or industry, was also extracted. In case of any missing relevant data, the authors of the included articles were contacted via email.

### 2.6 Synthesis of Results

The primary outcome was postoperative pain at the palatal donor site evaluated with a visual analog scale (VAS), dichotomic (i.e., presence/absence of pain), or with a surrogate outcome (e.g., intake of pain killers as number of pills or total amount of milligrams or number of days of intake). Secondary outcomes were assessments of wound healing at the donor site, sensitivity disturbances, and the effect of discomfort at the donor site during eating.

### 2.7 Statistical Analysis

A meta‐analysis was intended for RCT with (1) methodological homogeneity, (2) pain at the donor site recorded with VAS, and (3) comparing PIH with SIH. Study characteristics were summarized in tables, and quantitative syntheses were displayed using forest plots. Pairwise meta‐analysis was conducted using the software Review Manager 5.3, using a random‐effects model with inverse‐variance weighting to account for between‐study variability. Between‐study variance (*τ* ^2^) was estimated using the Restricted Maximum Likelihood (REML) method. Split‐mouth studies were analyzed using a paired‐data approach to account for the within‐patient correlation; a correlation coefficient of 0.5 was assumed when calculating the variance of the paired mean difference, unless reported otherwise. The resulting effect estimate was pooled with those from the parallel‐group trials. Statistical heterogeneity was assessed using the *I* ^2^ statistics, and the mean differences and 95% confidence intervals were calculated. Relevant and comparable timepoints as well as subgroup analyses due to different postoperative measures at the donor site were considered.

### 2.8 Risk of Bias

Two authors (R.E.L. and E.A.) evaluated the risk of bias (RoB) of the included studies independently; in case of disagreement, a third author was consulted (N.A.). The Cochrane Risk of Bias Tool (RoB 2.0) was used to evaluate RCT. The included studies were classified as having “low risk,” “some concerns,” or “high risk” for the following domains and overall (Higgins et al. 2011): (a) randomization process, (b) deviations from intended interventions, (c) missing outcome data, (d) measurement of the outcome, and (e) selection of the reported results. To evaluate the RoB of CT, the ROBINS‐I (Risk of Bias In Non‐randomized Studies of Interventions) was used (Sterne et al. 2016). Studies were classified as having “low,” “moderate,” “serious,” and “critical” RoB. Domains evaluated were (a) confounding, (b) selection of participants in the study, (c) classification of information, (d) deviation from intended interventions, (e) missing data, (f) measurements of outcomes, and (g) selection of reported outcomes.

### 2.9 Certainty of the Evidence Assessment

The certainty of the evidence for the primary outcome was assessed using the Grading of Recommendations Assessment, Development and Evaluation (GRADE) approach (Zhang et al. 2019) including a Summary of Findings (SoF) table. The certainty of the evidence was classified as high, moderate, low, or very low according to the GRADE domains of RoB, inconsistency, indirectness, imprecision, and publication bias.

## 3 Results

### 3.1 Study Selection and Characteristics

The flowchart of the study selection process is shown in Figure 1. A total of 8637 references were retrieved from the electronic search and, after removing 2094 duplicates, 6543 records were screened and 6531 removed based on title and abstract. Out of 12 studies that were read in full text, 7 articles were finally included (Zucchelli et al. 2010; Del Pizzo et al. 2002; Wessel and Tatakis 2008; Isler et al. 2018; Bakhishov et al. 2021; Beymouri et al. 2023; Lessa et al. 2025) (Table 1). Two studies were CTs (Wessel and Tatakis 2008; Lessa et al. 2025), and five studies were RCTs (Zucchelli et al. 2010; Del Pizzo et al. 2002; Isler et al. 2018; Bakhishov et al. 2021; Beymouri et al. 2023), of which one had a split‐mouth design (Beymouri et al. 2023), while the other RCT were performed in parallel group design. One of the CTs (Lessa et al. 2025) mentioned in the abstract a random group allocation; however, as the study was neither in the title nor in the text entitled as RCT and as additionally no randomization process was described, this study was judged as CT. The reasons for excluding five full‐texts (Harris 1997; Burkhardt et al. 2015; Pandit et al. 2016; Zangrando et al. 2021; Mashaly et al. 2022) are provided in Figure 1 and in Appendix Table A4. The agreement (Cohen's Kappa) between the two reviewers (N.A. and M.M.) at the abstract and full‐text level was 0.91 and 1, respectively.

**Figure 1:**
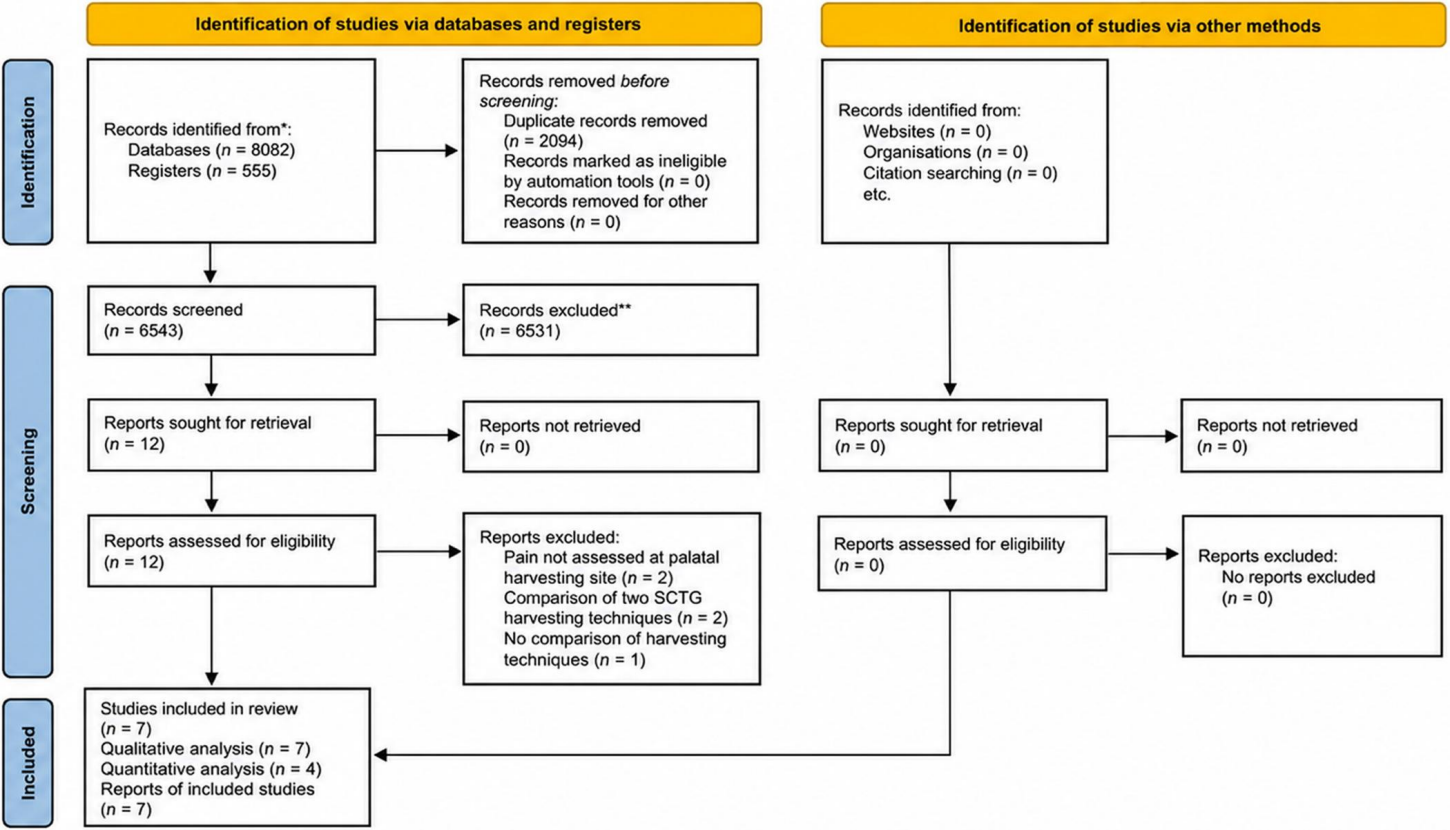
PRISMA flowchart.

**Table 1:** Characteristics of the included studies (*n* = 7).

Author year Study design Country	Type of graft (sites/group) Type of healing Incision technique^a^	Gender (female, %) Age (years, mean) Smoking status	Palatal mucosa thickness (mm) Donor region	Graft size (mm)			Postoperative care at the donor site Follow‐up (weeks)
				Thickness	Length (mesio‐distal)	Height (corono‐apical)	
Del Pizzo et al. (2002) RCT (parallel) Italy	S‐CTG (12) PIH SI	NR NR NS	NR PM	NR	12	8	NP 4
	S‐CTG (12) PIH TD		NR PM	NR	12	8	NP 4
	FGG (12) SIH		NR PM	NR	12	8	NP 4
Wessel and Tatakis (2008) CT (parallel) United States	S‐CTG (12) PIH PI or SI	50 36.6 NR	NR NR	NR	NR	NR	NP 3
	FGG (11) SIH	64 42.1 NR	NR NR	NR	NR	NR	Palatal stent 3
Zucchelli et al. (2010) RCT (parallel) Italy	S‐CTG (25) PIH TD	60 32.2 < 10 cig/day	3.1 (0.5) PM‐M	1.3 (0.2)	10.7 (0.8)	6.1 (0.8)	NP 1
	D‐FGG (25) SIH	52 34.2 < 10 cig/day	3.1 (0.5) PM‐M	1.3 (0.1)	10.9 (0.3)	6.2 (0.9)	Equine‐derived collagen 1
Isler et al. (2018) RCT (parallel) Turkey	S‐CTG (12) PIH SI	50 40.9 NS	4.1 (0.3) PM‐M	1.6 (0.1)	12.1 (1.0)	7.8 (0.4)	NP 3
	FGG (12) SIH	58.3 42.4 NS	4.0 (0.3) PM‐M	1.5 (0.1)	12.1 (0.8)	8.4 (0.5)	NP 3
Bakhishov et al. (2021) RCT (parallel) Turkey	S‐CTG (14) PIH SI	28 40.6 NS	3.0 (0.4) PM	1.3 (0.1)	17.5 (2.3)^b^	4.6 (0.5)	NP 4
	D‐FGG (13) SIH	61 43.5 NS	3.1 (0.4) PM	1.4 (0.1)	20.4 (3.5)^b^	4.6 (0.4)	NP 4
Beymouri et al. (2023) RCT (split‐mouth) Iran	S‐CTG (12) PIH TD	33 46 NS	3.2 (0.2) PM‐M	1.1 (0.1)	NR	NR	Periodontal pack 1
	D‐FGG (12) SIH		3.3 (0.2) PM‐M	1.1 (0.1)	NR	NR	Periodontal pack 1
Lessa et al. (2025) CT (parallel)	S‐CTG (8) PIH SI (with periosteum)	50 37 NS	NR	NR	NR	NR	NP 3
	D‐FGG (8) SIH		NR	ca. 1.5	NR	NR	NP 3

*Note:* All continuous data are expressed as mean (S.D.).

Abbreviations: Cig, cigarettes; CT, controlled trial; D‐FGG, de‐epithelialized free gingival graft; FGG, free gingival graft; M, molar; NP, no protection; NR, not reported; NS, non‐smokers; PI, parallel incision; PIH, primary intention healing; PM, premolar; RCT, randomized controlled trial; S‐CTG, subepithelial connective tissue graft; SI, single incision; SIH, secondary intention healing; TD, trap door incision.

^a^ Only relevant for S‐CTG.

^b^ Indicates significant differences between the groups.

### 3.2 Study Populations

Details on the study populations are reported in Table 1. The total number of patients included was 188, contributing to 200 donor sites; a S‐CTG with PIH was harvested from 107 sites and a FGG/D‐FGG with SIH from 93 sites. The individual study populations ranged from 12 (Beymouri et al. 2023) to 50 patients (Zucchelli et al. 2010), and the follow‐up time for the primary outcome parameter (pain) ranged from 1 week (Zucchelli et al. 2010; Beymouri et al. 2023; Lessa et al. 2025) to 4 weeks (Del Pizzo et al. 2002; Bakhishov et al. 2021). Five studies (Del Pizzo et al. 2002; Isler et al. 2018; Bakhishov et al. 2021; Beymouri et al. 2023; Lessa et al. 2025) included only non‐smokers, one study included patients smoking up to 10 cigarettes per day (Zucchelli et al. 2010), and one study did not report on the smoking status (Wessel and Tatakis 2008).

### 3.3 Characteristics of the Donor Site, Harvesting Technique, and Palatal Graft

The treatment groups of the included studies harvested either a S‐CTG with a split‐thickness flap approach (Zucchelli et al. 2010; Del Pizzo et al. 2002; Wessel and Tatakis 2008; Isler et al. 2018; Bakhishov et al. 2021; Beymouri et al. 2023; Lessa et al. 2025), a FGG (Del Pizzo et al. 2002; Wessel and Tatakis 2008; Isler et al. 2018), or a D‐FGG (Zucchelli et al. 2010; Bakhishov et al. 2021; Beymouri et al. 2023; Lessa et al. 2025). Regarding the split‐thickness flap approach, either a parallel incision (PI) (Wessel and Tatakis 2008), trap door (TD) (Zucchelli et al. 2010; Del Pizzo et al. 2002; Beymouri et al. 2023), or single incision (SI) technique (Del Pizzo et al. 2002; Wessel and Tatakis 2008; Isler et al. 2018; Bakhishov et al. 2021; Lessa et al. 2025) was used. Only one study harvested the graft explicitly with the periosteum, using a periosteal elevator (Lessa et al. 2025). The palatal donor site was primarily in the premolar region, extending to the mesial aspect of the first molar (Del Pizzo et al. 2002; Isler et al. 2018) or the distal aspect of the second molar (Beymouri et al. 2023), with an average thickness of the palatal mucosa, prior to harvesting, of 3–4 mm. The graft size was standardized in one study (Del Pizzo et al. 2002), while it was otherwise based on the needs of the recipient site and recorded intra‐operatively including thickness (pre‐ and post‐de‐epithelialization), length (mesio‐distal), and height (apico‐coronal) in mm (Zucchelli et al. 2010; Isler et al. 2018; Bakhishov et al. 2021; Beymouri et al. 2023; Lessa et al. 2025). The thickness, length, and height of the palatal graft varied approximately between 1 to 2, 10 to 20, and 4 to 8 mm, respectively. Three studies (Zucchelli et al. 2010; Wessel and Tatakis 2008; Beymouri et al. 2023) applied some kind of palatal protection for the postoperative period, that is, an equine‐derived collagen (Zucchelli et al. 2010) or a palatal stent (Wessel and Tatakis 2008) but only in the D‐FGG/FGG group, while one study applied a periodontal pack in both treatment groups (Beymouri et al. 2023). Postoperative care also included systemic antimicrobials in two studies (Del Pizzo et al. 2002; Beymouri et al. 2023), and chlorhexidine rinsing in all studies (Zucchelli et al. 2010; Del Pizzo et al. 2002; Wessel and Tatakis 2008; Isler et al. 2018; Bakhishov et al. 2021; Beymouri et al. 2023; Lessa et al. 2025). For details, see Table 1.

### 3.4 Reported Outcome Variables

The primary outcome parameter, palatal pain, was assessed either using a VAS (six studies [Zucchelli et al. 2010; Wessel and Tatakis 2008; Isler et al. 2018; Bakhishov et al. 2021; Beymouri et al. 2023; Lessa et al. 2025]) and/or as absence/presence of pain (three studies [Del Pizzo et al. 2002; Wessel and Tatakis 2008; Isler et al. 2018]), or indirectly via intake of pain killers in mg, number of pills, and/or number of days taking pills (six studies [Zucchelli et al. 2010; Wessel and Tatakis 2008; Isler et al. 2018; Bakhishov et al. 2021; Beymouri et al. 2023; Lessa et al. 2025]). These pain‐related parameters were assessed between 1 day and 4 weeks postoperatively (Table 2).

**Table 2:** Summary of the primary outcome, that is, postoperative pain (including the terms morbidity and discomfort) at the donor site.

Author year Study design	Pain assessment	Type of graft Type of healing Incision technique^a^	Evaluation timepoint^b^						
			1 day	2 days	3 days	1 week	2 weeks	3 weeks	4 weeks
Del Pizzo et al. (2002) RCT (parallel)	Absence of postoperative discomfort (% of patients)	S‐CTG PIH SI	NR			50^c^	92	100	100
		S‐CTG PIH TD				50^c^	92	92	100
		D‐FGG SIH				0^c^	67	100	100
Wessel and Tatakis (2008) CT (parallel)	Presence of postoperative pain (% of patients)	S‐CTG PIH PI or SI	NR		50^c^	NR		33	NR
		FGG SIH			90^c^			36	
	Pain VAS (0–10)	S‐CTG PIH PI or SI	NR		3.5 (1.8)	NR		1.6 (2.3)	NR
		FGG SIH			4.8 (1.2)			1.4 (2.0)	
	Pain killer intake (number of pills)	S‐CTG PIH PI or SI	8.6 (5.5)			12.5 (10.4)			NR
		FGG SIH	11.1 (3.2)			17.8 (10.6)			
	Pain killer intake (number of days)	S‐CTG PIH PI or SI	3.2 (2.5)						
		FGG SIH	4.9 (2.4)						
Zucchelli et al. (2010) RCT (parallel)	Discomfort VAS (0–10)	S‐CTG PIH TD	NR			2.7 (2.2)	NR		
		D‐FGG SIH				3.1 (2.0)			
	Pain killer intake (mg)	S‐CTG PIH TD	2016 (1896.4)				NR		
		D‐FGG SIH	1656 (1532.2)				NR		
Isler et al. (2018) RCT (parallel)	Pain VAS (0–10)	S‐CTG PIH SI (flurbiprofen spray)	1.6 (2.7)	NR	1.1 (2.2)	1.1 (2.0)	0.4 (1.3)	0.0 (0)	NR
		D‐FGG SIH (flurbiprofen spray)	2.3 (3.4)		1.4 (1.7)	0.6 (1.6)	0.0 (0)	0.0 (0)	
		S‐CTG PIH SI (placebo spray)	1.6 (2.6)	NR	1.2 (1.8)	1.2 (2.0)	0.7 (1.6)	0.0 (0)	NR
		D‐FGG SIH (placebo spray)	1.3 (1.7)		3.7 (3.4)	3.6 (2.8)	0.1 (0.3)	0.0 (0)	
	Pain killer intake (mg)	S‐CTG PIH SI (flurbiprofen spray	160 (47.6)				NR		
		D‐FGG SIH (flurbiprofen spray)	160 (56.2)						
		S‐CTG PIH SI (placebo spray)	200 (59.6)				NR		
		D‐FGG SIH (placebo spray)	310 (88.8)						
	No postoperative discomfort (% of patients)	S‐CTG PIH SI (flurbiprofen spray	NR					100	NR
		D‐FGG SIH (flurbiprofen spray)						100	
		S‐CTG PIH SI (placebo spray)	NR					100	NR
		D‐FGG SIH (placebo spray)						100	
Bakhishov et al. (2021) RCT (parallel)	Pain VAS (0–10)	S‐CTG PIH SI	3.5 (2.4)	1.8 (2.0)	1.4 (1.7)	0.8 (1.7)	0.1 (0.3)	NR	0.0 (0)
		D‐FGG SIH	3.8 (1.9)	1.7 (0.9)	1.3 (1.8)	0.6 (1.0)	0.1 (0.3)		0.0 (0)
	Pain killer intake (mg)	S‐CTG PIH SI	1989.4 (1918.8)						
		D‐FGG SIH	2968 (2167.7)						
	Discomfort VAS (0–10)	S‐CTG PIH SI	3.2 (3.0)	1.0 (1.5)	0.7 (1.0)	0.3 (0.8)	0.6 (2.1)	NR	0.0 (0)
		D‐FGG SIH	3.2(2.1)	1.4 (1.1)	1.3 (2.1)	0.4 (0.9)	0.0 (0)		0.0 (0)
Beymouri et al. (2023) RCT (split‐mouth)	Pain VAS (0–10)	S‐CTG PIH TD	NR		1.7 (1.1)	0.4 (0.5)^c^	NR		
		D‐FGG SIH			2.4 (1.4)	2.3 (2.2)^c^			
	Pain killer intake (number of pills)	S‐CTG PIH TD	4.8±3.0			1.8 (3.3)^c^ Total: 6.6 (5.6)^c^	NR		
		D‐FGG SIH	7.7 ± 3.8			5.6 (4.6)^c^ Total: 13.3 (5.5)^c^	NR		
Lessa et al. (2025) CT (parallel)	Pain VAS (0–10)	S‐CTG PIH SI (with periosteum)	NR			3.0 (2.5)^c^	NR		
		D‐FGGSIH				6.4 (3.2)^c^			
	Pain killer intake (number of pills)	S‐CTG PIH SI (with periosteum)	3.6 (4.8)^c^				NR		
		D‐FGG SIH	9.9 (8.3)^c^						

Abbreviations: CT, controlled trial; D‐FGG, de‐epithelialized free gingival graft; FGG, free gingival graft; mg, milligrams; NR, not reported; PI, parallel incision; PIH, primary intention healing; RCT, randomized controlled trial; S‐CTG, subepithelial connective tissue graft; SI, single incision; SIH, secondary intention healing; TD, trap door incision; VAS, visual analog scale.

^a^ Only relevant for S‐CTG.

^b^ All continuous data are expressed as mean (S.D.).

^c^ Indicates significant differences between the groups.

The secondary outcome parameters were complete epithelialization (CE) (Del Pizzo et al. 2002), sensitivity disturbances (Del Pizzo et al. 2002; Bakhishov et al. 2021), effect on eating habits (Zucchelli et al. 2010; Del Pizzo et al. 2002; Isler et al. 2018), inability to chew (Zucchelli et al. 2010), stress (Zucchelli et al. 2010), and burning sensation (Bakhishov et al. 2021) (Table 3). A sensitivity disturbance was defined by Del Pizzo et al. (2002) as an objective sensory loss, recorded using a rubbing movement and a pin‐pressure nociception. The absence of a sensitivity disturbance was considered in this review as “normal sensitivity.” Any effect on eating habits was evaluated either as the percentage of patients maintaining normal eating patterns (Del Pizzo et al. 2002) or using a VAS (0: no change; 10: extreme change) (Zucchelli et al. 2010; Isler et al. 2018). Postoperative stress was defined in one study as the level of apprehension and fear regarding potential palatal wound disruption measured with a VAS (Zucchelli et al. 2010).

**Table 3:** Summary of the secondary outcomes.

Author year Study design	Type of graft Type of healing Incision technique^a^	Effect on eating habits	Wound healing	Normal sensitivity	Stress (VAS)	Burning sensation (VAS)
Del Pizzo et al. (2002) RCT (parallel)	S‐CTG PIH SI	NEH (% of patients) 1w: 42 2w: 100 3w: 100 4w: 100	% of patients with CE 1w: 8 2w: 50 3w: 100^b^ 4w: 100	% of patients 1w: 8 2w: 25 3w: 42 4w: 66 6w: 92 8w: 100	NR	NR
	S‐CTG PIH TD	NEH (% of patients) 1w: 42 2w: 75 3w: 100 4w: 100	% of patients with CE 1w: 8 2w: 33 3w: 92 4w: 100	% of patients 1w: 8 2w: 8 3w: 33 4w: 66 6w: 84 8w: 100		
	D‐FGG SIH	NEH (% of patients) 1w: 16 2w: 67 3w: 92 4w: 100	% of patients with CE 1w: 0 2w: 16 3w: 50^b^ 4w: 100	% of patients 1w: 0 2w: 0 3w: 16 4w: 50 6w: 75 8w: 100		
Zucchelli et al. (2010) RCT (parallel)	S‐CTG PIH TD	ITC (VAS) 1w: 2.0 (1.9)^b^	NR	NR	2.1 (1.3)^b^	NR
	D‐FGG SIH	ITC (VAS) 1w: 3.9 (2.0)^b^			4.5 (1.5)^b^	
Isler et al. (2018) RCT (parallel)	S‐CTG PIH SI (flurbiprofen spray)	CDH (VAS) 1d: 6.8 (2.8) 3d: 3.9 (2.3) 1w: 0.9 (2.2) 2w: 1.0 (0.9) 3w: 0.3 (0.7)	% of patients with CE 1w: 0% 2w: 33% 3w: 66% 4w:100% 6w: 100% 8w: 100%	NR	NR	1d: 0.9 (2.2) 3d: 1.2 (1.7) 1w: 0.8 (1.8) 2w: 0.1 (0.3) 3w: 0.0 (0)
	D‐FGG SIH (flurbiprofen spray)	CDH (VAS) 1d: 3.0 (3.6) 3d: 1.1 (1.6) 1w: 0.8 (1.4) 2w: 0.0 (0) 3w: 0.0 (0)	% of patients with CE 1w: 0% 2w: 25% 3w: 33% 4w: 50% 6w: 100% 8w: 100%			1d: 1.2 (2.6) 3d: 1.3 (1.7) 1w: 1.2 (1.8) 2w: 0.0 (0) 3w: 0.0 (0)
	S‐CTG PIH SI (placebo spray)	CDH (VAS) 1d: 6.2 (2.6) 3d: 4.0 (2.0) 1w: 1.0 (1.8) 2w: 0.4 (1.6) 3w: 0.0 (0) 4w: 0.0 (0)	% of patients with CE 1w: 0% 2w: 50% 3w:75% 4w: 100% 6w: 100% 8w: 100%	NR	NR	1d: 0.8 (1.8) 3d: 0.6 (0.9) 1w: 0.6 (1.5) 2w: 0.4 (1.3) 3w: 0.0 (0)
	D‐FGG SIH (placebo spray)	CDH (VAS) 1d: 5.5 (2.0) 3d: 4.7 (2.5) 1w: 3.9 (3.0) 2w: 0.3 (0.7) 3w: 0.0 (0) 4w: 0.0 (0)	% of patients with CE 1w: 0% 2w: 25% 3w: 66% 4w: 75% 6w: 100% 8w: 100%			1d: 0.5 (1.6) 3d: 1.9 (2.9) 1w: 2.4 (2.3) 2w: 0.6 (1.3) 3w: 0.0 (0)
Bakhishov et al. (2021) RCT (parallel)	S‐CTG PIH SI	NR	NR	VAS 1d: 2.6 (2.3) 2d: 1.7 (1.9) 3d: 1.2 (1.9) 1w: 0.6 (1.3) 2w: 0.3 (0.8) 4w: 0.1 (0.3)	NR	NR
	D‐FGG SIH			VAS 1d: 2.1 (2.6) 2d: 0.9 (1.2) 3d: 0.9 (1.4) 1w: 0.5 (0.9) 2w: 0.3 (0.9) 4w: 0.0 (0)		

*Note:* All continuous data are expressed as mean (S.D.).

Abbreviations: CDH, changes in dietary habits; CE, complete epithelialization; d, days; D‐FGG, de‐epithelialized free gingival graft; ITC, inability to chew; NEH, normal eating habits; NR, not reported; PIH, primary intention healing; S‐CTG, subepithelial connective tissue graft; SI, single incision; SIH, secondary intention healing; TD, trap door incision; VAS, visual analog scale; w, weeks.

^a^ Only relevant for S‐CTG.

^b^ Indicates significant differences between the groups.

### 3.5 Summary of the Results—Primary Outcome Parameter

The results regarding the primary outcome parameter palatal pain are summarized in Table 2. Statistically significant differences were reported in four studies for at least one of the parameters assessing pain at the harvesting site (Del Pizzo et al. 2002; Wessel and Tatakis 2008; Beymouri et al. 2023; Lessa et al. 2025). Del Pizzo et al. (2002) reported a significantly higher proportion of patients reporting no postoperative discomfort from the donor site after 1 week in both groups aiming for PIH (i.e., 50% each) compared to the group resulting in SIH (i.e., 0%). Similarly, Wessel and Tatakis (Wessel and Tatakis 2008) observed a significant difference between the groups with either PIH or SIH, regarding the proportion of patients experiencing postoperative palatal pain after 3 days, that is, 50% in the PIH group versus 90% in the SIH group. Beymouri et al. (2023) detected a significant difference in the pain VAS scores after 1 week, that is, 0.4 (0.5) in the PIH group compared to 2.3 (2.2) in the SIH group, which in turn resulted also in a significant lower pain killer consumption within the first week in the PIH compared to the SIH group, that is, 1.8 (3.3) versus 5.6 (4.6) pills, respectively. Finally, Lessa et al. (2025) reported significantly higher pain VAS scores and significantly higher analgesic consumption in the SIH group after 1 week. The remaining three studies could not detect a statistically significant difference regarding pain at the donor site between the groups. However, it should be noted that Isler et al. (2018) did not present a statistical comparison between the PIH and SIH groups.

### 3.6 Summary of the Results—Secondary Outcome Parameters

The results of the secondary outcome parameters are summarized in Table 3. They have been reported in four studies (Zucchelli et al. 2010; Del Pizzo et al. 2002; Isler et al. 2018; Bakhishov et al. 2021), and significant differences between the groups were recorded in two studies. Del Pizzo et al. (2002) reported a significantly higher rate of CE after 3 weeks in the PIH group with SI technique compared to the SIH group, that is, CE was achieved in 100% versus 50% of the patients, respectively. Zucchelli et al. (2010) reported significantly higher VAS values in the SIH group compared to the PIH group regarding inability to chew and stress 1 week postoperatively. The remaining two studies could not detect a statistically significant difference between the groups. However, it should be noted that Isler et al. (2018) did not present a statistical comparison between the PIH and SIH groups.

### 3.7 Synthesis of the Results

Four RCTs evaluated palatal pain using a VAS (Zucchelli et al. 2010; Isler et al. 2018; Bakhishov et al. 2021; Beymouri et al. 2023) and were summarized in a meta‐analysis (Figure 2). No statistically significant differences in terms of pain perception were observed between the PIH and SIH groups at 3 days postoperatively (three studies; effect size: 0.73 [95% CI: –0.32 to 1.78, *p* = 0.17]; no significant heterogeneity among the studies [*I* ^2^ = 57%; *p* = 0.10]). Similarly, no statistically significant differences were observed between the groups at 7 days postoperatively (four studies; effect size: 1.03 [95% CI: –0.12 to 2.17, *p* = 0.08]; statistically significant heterogeneity among the studies [*I* ^2^ = 70%; *p* = 0.02]). A subgroup analysis including only studies without palatal wound protection was conducted. Neither at 3 days nor at 7 days postoperatively, any significant difference was detected based on the data of two studies; for details, see Figure 2. A subgroup analysis including only studies using a palatal wound protection was not possible as only a single study was available.

**Figure 2:**
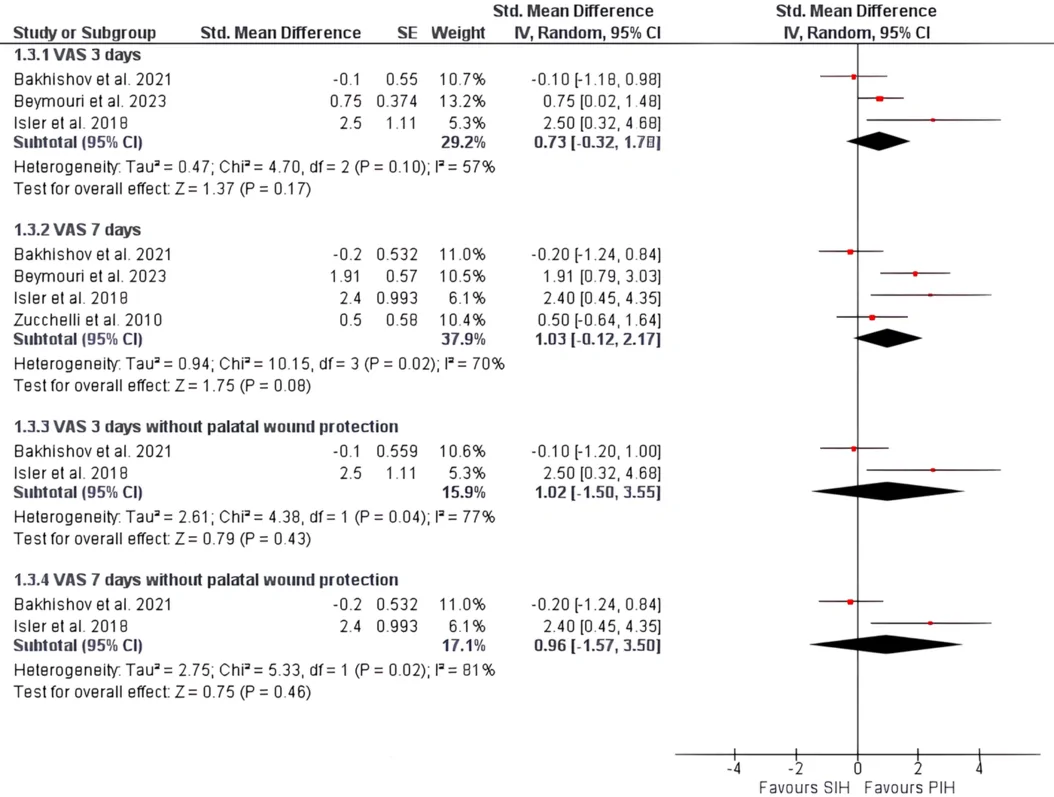
Pairwise meta‐analyses for the primary outcome parameter pain perception (VAS) at the palatal donor site after 3 and 7 days; additionally, a subgroup analysis including only studies without palatal wound protection was performed. Please note that if one of the groups (PIH or SIH) was favored, this indicates that less pain occurred.

Due to the limited number of studies included in the meta‐analysis, assessment of publication bias and small‐study effects was not performed.

### 3.8 RoB, Certainty of the Evidence, and Funding

RoB assessment of the five RCTs and two CTs is presented in Figures 3 and 4, respectively. Three RCTs (Zucchelli et al. 2010; Del Pizzo et al. 2002; Bakhishov et al. 2021) were rated with “some concerns” and two with low RoB (Isler et al. 2018; Beymouri et al. 2023). The CT were rated as having moderate overall RoB (Wessel and Tatakis 2008; Lessa et al. 2025). The certainty of the evidence was judged to be very low, mainly explained by the RoB, inconsistency, and imprecision among the included studies (Table 4). Most studies were funded by the authors' institutions, with only two receiving external support, either from a pharmaceutical company (Isler et al. 2018) or through a university research grant (Bakhishov et al. 2021) (Appendix Table A5).

**Figure 3:**
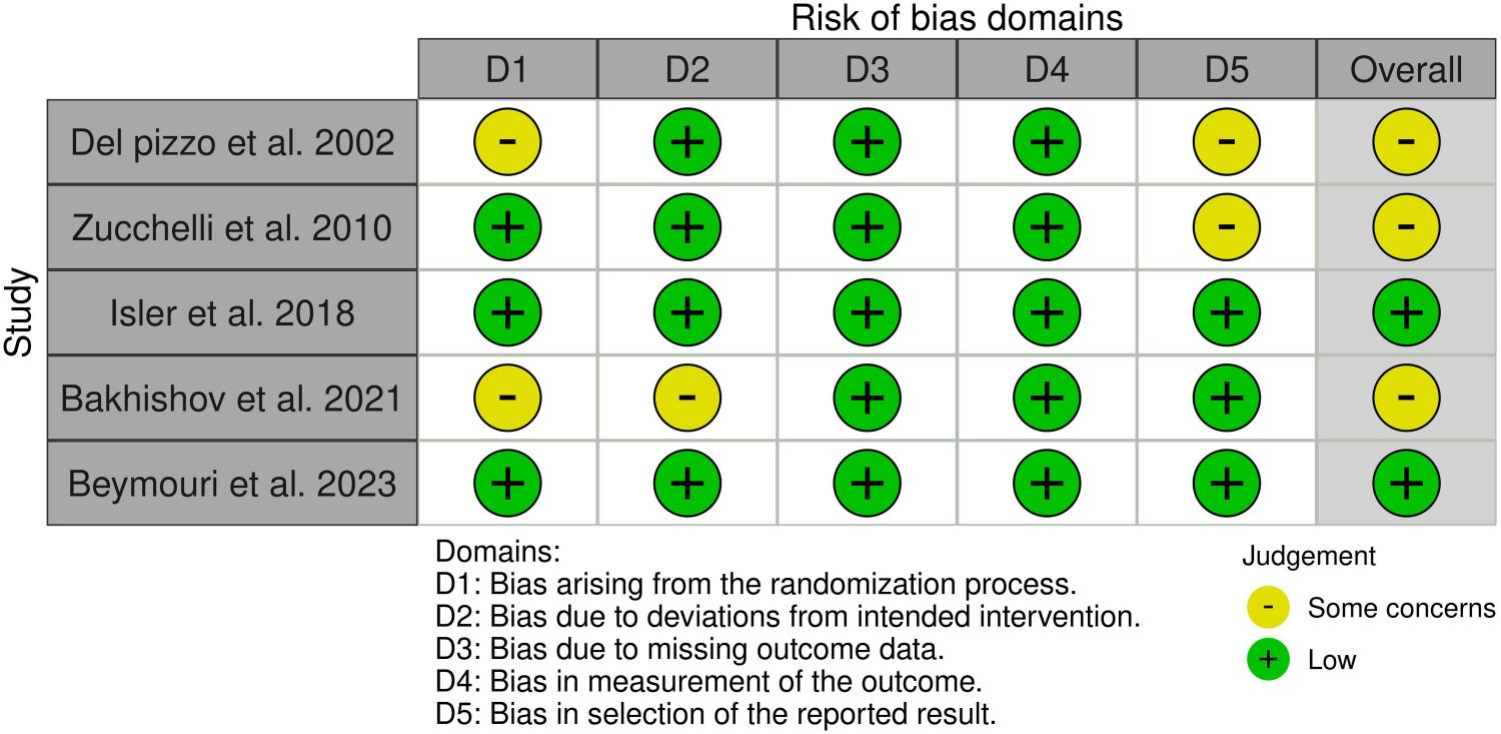
Traffic‐light plot of the RoB2 assessment of the included RCT.

**Figure 4:**
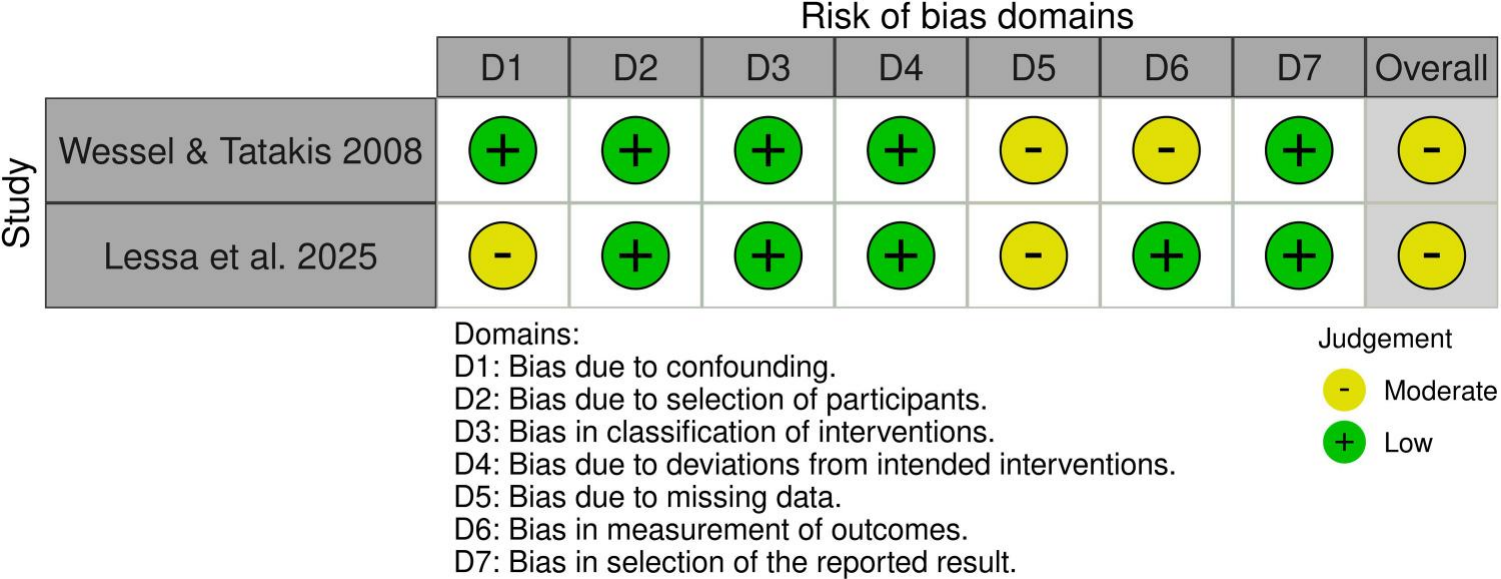
Traffic‐light plot of the ROBINS‐I assessment of the included CT.

**Table 4:** Summary of findings for the primary outcome parameter pain perception (VAS).

Outcome	Participants (studies)	Mean difference (95% CI)	Certainty of evidence (GRADE)	Reasons for downgrading
Pain perception (VAS) at 3 days	75 participants (3 RCTs)	MD 0.73 higher (−0.32‐1.78)	⊕◯◯◯ Very low	ROB, inconsistency, imprecision
Pain perception (VAS) at 7 days	125 participants (4 RCTs)	MD 1.03 higher (−0.12‐2.17)	⊕◯◯◯ Very low	ROB, inconsistency, imprecision

*Note:* Higher values indicate greater postoperative pain.

Abbreviations: CI, confidence interval; MD, mean difference; RCT, randomized controlled trial; VAS, visual analog scale.

## 4 Discussion

This systematic review synthesized the available evidence from CT and RCT assessing pain perception at the palatal donor site after two different graft harvesting techniques, resulting either in PIH or SIH. Out of seven studies (Zucchelli et al. 2010; Del Pizzo et al. 2002; Wessel and Tatakis 2008; Isler et al. 2018; Bakhishov et al. 2021; Beymouri et al. 2023; Lessa et al. 2025) meeting the inclusion criteria, four studies including both CT (Del Pizzo et al. 2002; Wessel and Tatakis 2008; Beymouri et al. 2023; Lessa et al. 2025) indicated significant less pain perception when the grafts were collected with a technique aiming for PIH, while meta‐analysis on RCT failed to identify significant differences between PIH and SIH approaches, irrespective of whether a postoperative palatal wound protection was used or not.

The observed lack of difference in postoperative pain perception between the two surgical harvesting methods may, at least partly, be attributed to differences in postoperative wound care among the included studies and to the overall limited number of available RCT. Specifically, in two of the studies included in the meta‐analysis, palatal wound protection was used, either in both treatment groups (Beymouri et al. 2023) or only in the D‐FGG group (Zucchelli et al. 2010), while the two other studies did not use any wound protection. This obviously introduces some bias, which is difficult to estimate/weight, especially as the subgroup analysis was limited to two studies and only possible for studies not using palatal wound protection. Nevertheless, the study from Zucchelli et al. which used palatal wound protection only in the D‐FGG group, clearly contributed to a lack of difference in pain perception between the PIH and SIH approaches at 7 days. Additionally, both CTs, which were not included in the meta‐analysis, showed a significant difference regarding pain favoring the PIH group, that is, more pain was noted for the SIH approach. In this context, different measures to protect the palatal wound have been proposed and have met varying degrees of success; although there is no gold standard established (Silva et al. 2023; Leite et al. 2025), it may be suggested that using some kind of wound protection of the open palatal wound after harvesting a FGG/D‐FGG should be considered as standard of care to minimize postoperative patient morbidity (Silva et al. 2023; Leite et al. 2025). In this context, pain following palatal graft harvesting appears to be more pronounced during the first postoperative days and generally decreases rapidly thereafter. Therefore, assessment at early timepoints is likely to provide the clinically most relevant information when comparing harvesting techniques, whereas later evaluations may fail to reflect acute postoperative morbidity. However, variability in the exact timepoints assessed often limited direct comparisons among the included studies. Future investigations should adopt standardized and frequent pain assessment during the first postoperative week to improve comparability for future evidence synthesis.

Besides the surgical technique per se to harvest a graft, several other factors may influence postoperative pain. These factors include, but are not limited to, the surgical time, the graft size/dimensions, the remaining palatal tissue thickness, and the occurrence of postoperative palatal flap necrosis. For example, it was reported that patients with palatal flap necrosis or a flap dehiscence and SIH instead of PIH after S‐CTG harvesting consumed more analgesics compared to patients with uneventful healing after harvesting a D‐FGG (with intentionally SIH) (Zucchelli et al. 2010). Further, a negative correlation has been previously found between residual palatal tissue thickness and use of analgesics, that is, patients with a thinner remaining tissue at the palate after graft harvesting, consumed significantly more analgesics (Zucchelli et al. 2010), and indeed, the D‐FGG harvesting technique regularly results in a thicker residual palatal tissue. Lastly, a systematic review further suggested that graft dimensions of epithelialized grafts are positively associated with pain, that is, thicker/larger grafts (thickness > 2 mm; mesio‐distal length ≥ 14 mm) were associated with higher pain levels compared to smaller grafts (Escobar et al. 2022). However, these factors were often not considered in the original studies included herein, and thus it was impossible to evaluate their contribution in pain perception between the two surgical techniques.

Previous reports have indicated that using a soft tissue graft collected as D‐FGG may—apart from easier handling (Zucchelli and Mounssif 2015; Zucchelli et al. 2020)—provide superior outcomes at the recipient site compared to those achieved with a S‐CTG. In particular, a systematic review on this topic concluded that CAF at natural teeth in combination with a D‐FGG leads to superior outcomes compared to CAF using a S‐CTG; significant differences in favor of a D‐FGG were observed for mean root coverage, recession reduction, KT gain, probing pocket depth reduction, and CAL (Tavelli et al. 2019). These clinical differences between the two types of grafts may be attributed to their distinct tissue composition (Silva et al. 2023; Leite et al. 2025; Escobar et al. 2022; Zucchelli and Mounssif 2015); in particular, in a histomorphometric study (Bertl et al. 2015), higher proportions of fibrous connective tissue and lower proportions of fatty/glandular tissue were observed in grafts collected as D‐FGG compared to grafts collected as S‐CTG, which in turn may result in the greater dimensional stability observed for D‐FGG over time.

It should also be noted that the secondary outcome parameters assessed herein displayed a significant difference in favor of PIH approaches in two out of four studies (Zucchelli et al. 2010; Sterne et al. 2016). Specifically, one study (Zucchelli et al. 2010) reported significantly higher inability to chew and higher stress levels among patients in the SIH group compared to patients in the PIH group. The second study (Del Pizzo et al. 2002) reported a significantly higher CE rate in the PIH compared to the SIH group after 3 weeks. The same parameter, that is, percentage of patients achieving CE, was assessed also in another study (Isler et al. 2018), which also showed advantages for the PIH compared to the SIH groups. However, this specific study did not present a statistical comparison between the PIH and SIH groups, but only between different treatment modalities, that is, flurbiprofen versus placebo spray. Nevertheless, considering the obvious differences in the residual wound between harvesting either a S‐CTG or a D‐FGG, a difference in the CE rate comes without surprise.

When interpreting the results of this synthesis of the available evidence on this topic, it should be kept in mind that it is suffering by the limited number of eligible trials, the different follow‐up periods, and the inconsistency regarding the reporting of factors that may influence post‐operative pain (e.g., analgesic regimens, use of palatal wound protection, residual palatal tissue thickness, graft dimension, and smoking status). Furthermore, the subgroup analysis was limited to two studies and should be considered exploratory, while the limited number of available studies did not allow to reliably assess any publication bias and small‐study effect.

## 5 Conclusions

- Current evidence does not confirm that aiming for PIH when harvesting a palatal soft tissue graft causes significantly less pain at the donor site compared to a technique involving SIH. Nevertheless, this lack of significance might be, at least partly, due to the variability among the included studies, for example, regarding graft/wound dimensions, and the overall limited number of available RCT, especially when assessing separately studies with or without the use of palatal wound protection.
- Only a single study reported significantly higher inability to chew and higher stress levels among patients of the SIH group compared to patients of the PIH group.
- A single study reported significantly superior wound healing (i.e., faster CE) after using an approach aiming for PIH compared to an approach with SIH; however, this comes without surprise.
- Future RCT should include larger sample sizes, standardized graft harvesting techniques including a detailed reporting of graft dimensions, harvesting depth, and residual palatal tissue thickness, standardized postoperative analgesic protocols and donor‐site management, and validated pain assessment tools at relevant postoperative timepoints.

## Author Contributions

**Natalia Asquino:** study idea, literature search, data collection, data analysis, data interpretation, manuscript drafting. **Rafael Esteves Lima:** literature search, data collection, data analysis, data interpretation, manuscript drafting. **Ernesto Andrade:** data collection, data interpretation, manuscript revision. **Adriana Drescher:** data collection, data interpretation, manuscript drafting. **Magdalena Mayol:** data collection, literature search, data interpretation, manuscript drafting. **Kristina Bertl:** data analysis, data interpretation, manuscript drafting. **Andreas Stavropoulos:** data interpretation, manuscript revision.

## Funding

The authors have nothing to report.

## Conflicts of Interest

All authors declare to have no conflict of interest in relation to this submission. Andreas Stavropoulos is Editor in Chief and Kristina Bertl is Associate Editor of the journal *Clinical and Experimental Dental Research*.

## Data Availability

The data are available from the corresponding author upon reasonable request.

## Appendix A

Tables A1, A2, A3

**Table A1:** Search strategy for PubMed (last search on February 28, 2026, 3191 hits).

Search number	
1	Connective OR graft OR connective tissue graft OR free gingival graft OR de‐epithelized free gingival graft OR de‐epithelialized free gingival graft OR de‐epithelized OR de‐epithelialized OR deepithelialized OR soft tissue graft OR gingival autograft OR soft tissue augmentation OR soft tissue transplantation OR connective tissue [MeSH Terms] OR tissue and organ harvesting [MeSH Terms]
2	Palate [MeSH Terms] OR palat*
3	Pain OR discomfort OR morbidity
4	Analgesics [MeSH Terms] OR analgesic intake
5	Bleeding
6	Eating AND (difficulties OR disturbance*)
7	Patient Reported Outcome Measures [MeSH Terms] OR PROM* OR patient reported outcome measure* OR patient‐reported outcome measure* OR patient reported OR patient‐reported
8	#3 OR #4 OR #5 OR #6 OR #7
9	#1 AND #2 AND #8

**Table A2:** Search strategy for Embase (last search on February 28, 2026, 3760 hits).

#4	#1 AND #2 AND #3
#3	Pain OR discomfort OR morbidity OR “analgesic intake” OR “analgesic agent”/exp OR bleeding OR “eating disturbance*” OR “patient‐reported outcome”/exp OR prom* OR “patient reported outcome measure*” OR “patient‐reported outcome measure*” OR “patient reported” OR “patient‐reported”
#2	Palat* OR “palate”/exp
#1	Connective OR “graft”/exp OR graft OR “connective tissue graft”/exp OR “connective tissue graft” OR “free gingival graft”/exp OR “free gingival graft” OR “de‐epithelized free gingival graft” OR “de‐epithelialized free gingival graft” OR “de‐epithelized” OR “de‐epithelialized” OR deepithelized OR deepithelialized OR “soft tissue graft”/exp OR “soft tissue graft” OR “gingival autograft” OR “soft tissue augmentation”/exp OR “soft tissue augmentation” OR “soft tissue transplantation” OR “graft harvesting”/exp OR “graft harvesting” OR “connective tissue”/exp OR “connective tissue”

**Table A3:** Search strategy for CENTRAL (last search on February 28, 2026, 561 hits).

ID	Search
#1	MeSH descriptor: [Patient Reported Outcome Measures] explode all trees
#2	(PROM* OR patient reported outcome measure* OR patient‐reported outcome measure* OR patient reported OR patient‐reported)
#3	(#1 OR #2)
#4	(Eating AND (difficulties OR disturbance*))
#5	(Bleeding)
#6	MeSH descriptor: [Hemorrhage] explode all trees
#7	#5 OR #6
#8	MeSH descriptor: [Analgesics] this term only
#9	(Analgesic intake)
#10	#8 OR #9
#11	(Pain OR discomfort OR morbidity)
#12	MeSH descriptor: [Palate] explode all trees
#13	(Palat*)
#14	#12 OR #13
#15	Connective OR graft OR connective tissue graft OR free gingival graft OR de‐epithelized free gingival graft OR de‐epithelialized free gingival graft OR de‐epithelized OR de‐epithelialized OR deepithelized OR deepithelialized OR soft tissue graft OR gingival autograft OR soft tissue augmentation OR soft tissue transplantation
#16	MeSH descriptor: [Connective Tissue] this term only
#17	MeSH descriptor: [Tissue and Organ Harvesting] explode all trees
#18	#15 OR #16 OR #17
#19	#3 OR #4 OR #7 OR #10 OR #11
#20	#14 AND #18 AND #19

**Table A4:** Studies excluded after full‐text evaluation and reason for exclusion.

Author (year)	Reason for exclusion
Harris (1997)	Comparison of 2 S‐CTG harvesting techniques (i.e., trap door vs. parallel incisions)
Burkhardt et al. (2015)	No comparison of different graft harvesting techniques
Pandit et al. (2016)	Comparison of 2 S‐CTG harvesting techniques (i.e., trap door with unigraft knife vs. Langer & Langer)
Zangrando et al. (2021)	Pain was not specifically assessed at the palatal harvesting site
Mashaly et al. (2022)	Pain was not specifically assessed at the palatal harvesting site

Abbreviation: S‐CTG, subepithelial connective tissue graft.

**Table A5:** Summary of the available information on funding of the included studies.

Author (year)	Funding
Wessel and Tatakis (2008)	Authors' institution
Del Pizzo et al. (2002)	Authors' institution
Zucchelli et al. (2010)	Authors' institution
Isler et al. (2018)	Sanovel Pharmaceuticals (Istanbul, Turkey) provided the fluribuprofen spray and a placebo product
Bakhishov et al. (2021)	Gazi University Scientific Research Projects coordination unit (grant number 03/2018‐16)
Beymouri et al. (2023)	Authors' institution
Lessa et al. (2025)	Authors' institution
